# Clinical and genetic analysis of a Chinese family with GM1 gangliosidosis caused by a novel mutation in GLB1 gene

**DOI:** 10.3389/fped.2025.1507098

**Published:** 2025-01-20

**Authors:** Biao Zhang, Xiao-li Huang, Xin-xin Lu, Heng-bin Huang, Yan-an Wu

**Affiliations:** ^1^ Department of Clinical Laboratory, Shengli Clinical Medical College of Fujian Medical University, Fujian Provincial Hospital, Fuzhou University Affiliated Provincial Hospital, Fuzhou, China; ^2^ Center of Clinical Laboratory, School of Medicine, Zhongshan Hospital of Xiamen University, Xiamen University, Xiamen, China

**Keywords:** GM1 gangliosidosis, GLB1 gene, β-galactosidase, heterozygous mutation, whole exon sequencing

## Abstract

**Objective:**

To describe the clinical presentation and novel mutation in the ganglioside-beta-galactosidase gene (GLB1) gene in a Chinese family with GM1 gangliosidosis.

**Methods:**

We collected clinical data from a Chinese family with GM1 gangliosidosis, and performed whole exon sequencing (WES) of the proband and his parents. The pathogenic sites of candidate genes were targeted, and the detected exon mutations were verified by Sanger sequencing. Enzyme activity of β-galactosidase was detected in 293T cells transiently transfected with plasmids encoding the detected mutations.

**Results:**

Two siblings in this family presented with neurological degeneration, and were classified as the late-infantile type. Two siblings and their parents underwent WES of the peripheral blood. A reported missense mutation c.446C>T and a novel mutation c.1058_1059delinsAA in GLB1 gene inherited from the mother and father respectively were identified. The mutant c.1058_1059delinsAA retained β-galactosidase activity at 0% of wild-type GLB1.

**Conclusion:**

This study identified a novel mutation of the GLB1 gene in a Chinese family with GM1 gangliosidosis and provided new insights into the molecular characteristics and genetic counseling of GM1 gangliosidosis.

## 1 Introduction

GM1 gangliosidosis is an autosomal recessive lysosomal storage disorder caused by mutations in the GLB1 gene, leading to a deficiency of acid β-galactosidase (1, 2). This enzyme deficiency results in the accumulation of glycolipids, sulfated keratin, and GM1 gangliosides in certain tissues, especially in neurons of the peripheral and central nervous systems (3), since gangliosides are particularly abundant in the neuronal plasma membrane (4). This accumulation leads to neuronal cell death and progressive central nervous system dysfunction, with varying severity and age at onset (5). GM1 gangliosidosis is a rare disorder with an incidence of approximately 1:100,000–1:200,000 newborns (6).

According to age of onset and severity of central nervous system involvement, this disease is usually divided into three clinical phenotypes: infantile, late-infantile/juvenile and adult. Type I or infantile form, which is the most severe and frequent form,has an early onset and usually starts at birth. Its manifestations are abnormal face, convulsion, severe intellectual disability, impaired vision and hearing (7). Most children die of convulsions, recurrent respiratory infections and paroxysmal tachycardia before the age of 2 years. The onset time of type II or late-infantile form is from 7 months to 3 years. It presents with systemic central nervous system involvement, accompanied by psychomotor deterioration, seizures, localized skeletal involvement, and childhood survival (7). Usually, there is no hepatosplenomegaly and cherry red spots. The onset age of type III or adult form is 3–30 years old. The symptoms of this type are lighter than those of type I and type II, showing obvious individual differences. Some patients only have focal neurological signs such as dystonia, while some patients more seriously involve extravertebral signs and intellectual disability (8, 9).

The GLB1 gene is located on 3p21.33 and consists of 16 exons (10). Its encoded protein is responsible for the breakdown of GM1 ganglioside in nerve cells. Mutations in this gene are the sole genetic factors for various forms of GM1 gangliosidosis. To date, over 280 mutations have been documented as pathogenic or likely pathogenic in the GLB1 gene on the Clinvar website (ncbi.nlm.nih.gov/clinvar). The majority of these mutations are classified as missense, often observed in compound heterozygous states (7), resulting in proteins with markedly variable enzymatic activities and clinical phenotypes that are similarly variable (10, 11). The residual activity of β-galactosidase depends on the position and nature of the mutated nucleotide, thus determining the severity of the disease. Individuals with higher β-galactosidase activity usually have a later age of onset and milder signs and symptoms (12). Therefore, GLB1 mutation analysis is necessary for infantile patients with lysosomal storage disease or neurological degeneration.

Owing to their high efficiency and cost-effectiveness, new technologies such as whole exome sequencing can expedite disease diagnosis and prevent futile assessments. Consequently, they are increasingly being applied in genetic disease diagnosis and, when feasible, therapeutic interventions are implemented for patients. In this study, we examined GLB1 mutations in two patients from a Chinese Han family with GM1 gangliosidosis. A previously unreported novel mutation was identified in these patients. We assessed the influence of the identified mutation on the clinical phenotype through whole exome sequencing and β-galactosidase activity assays, which will help us provide genetic counseling.

## 2 Patients and methods

### 2.1 Patients

**Figure 1:**
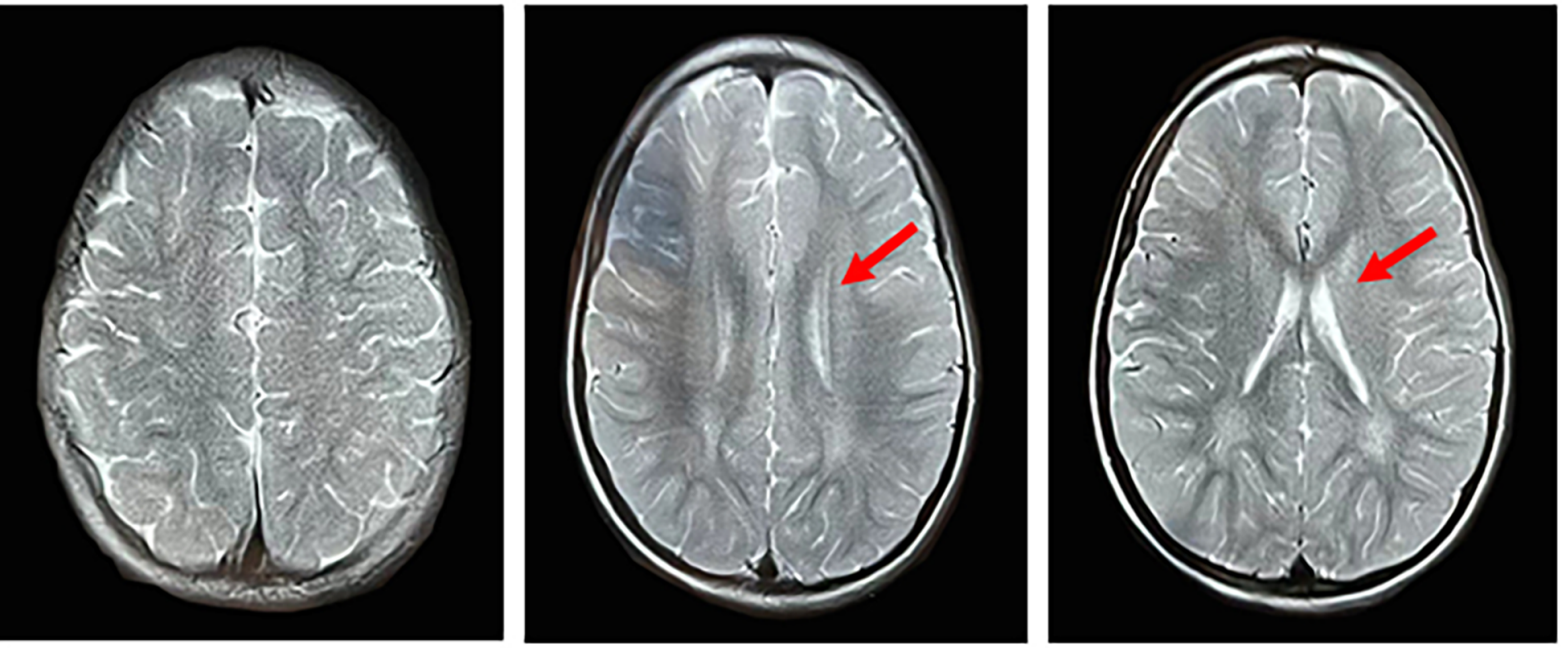
MRI image of the proband. It indicates symmetrical abnormal signals in the white matter area around the bilateral ventricles, mainly around the occipital horns. The lesions showed long T1, long T2 signals and FLAIR high signals.

The male patient, aged 10 years and 2 months, was admitted for progressive intellectual and motor regression. The patient's cognitive and motor skills were normal until the age of 1 year and 6 months. Subsequently, he experienced cognitive degeneration, decreased mobility, unsteady gait, occasional falls, and intermittent seizures. At the age of 2 years and 8 months, he was admitted to the local hospital. MRI revealed symmetric abnormalities in the white matter surrounding the bilateral ventricles, particularly around the occipital horns, suggesting potential cerebral white matter dysplasia (Figure 1). The video electroencephalogram indicated a significant slowing of background activity, accompanied by a marked increase in slow-wave activity during sleep. Sleep was characterized by a predominance of medium to high amplitude 2.5–3 Hz sharp (spike) slow waves, especially in the bilateral frontal and anterior temporal regions, with a more pronounced right-sided involvement. The echocardiogram revealed no significant abnormalities. In the laboratory tests, aside from mild elevations in blood ammonia and lactate, all other metabolic and hematological parameters remained within the normal range. The patient was bedridden, unable to recognize faces, and lacked conscious response to external stimuli. Based on onset age, disease progression and genetic analysis, the patient was diagnosed with the late-infantile form of GM1 gangliosidosis. The parents exhibited normal physical characteristics and are not first-degree relatives. They denied any family history of hereditary diseases.

The patient's older brother presented with the late-infantile form of the disease, had similar clinical manifestations including delayed motor and cognitive development, muscle weakness, spinal deformities and neurodevelopmental degeneration. The brother died of severe pulmonary infections and seizures at 13 years of age.

This study fully complied with the Tenets of the Declaration of Helsinki, and was approved by the Ethics Committee of the Fujian provincial hospital (Fuzhou, Fujian, China). Informed consent was obtained from the subject's parents before testing.

### 2.1 Methods

#### 2.1.1 Genomic DNA extraction

Genomic DNA was extracted from peripheral blood samples using Blood Genomic DNA Mini Kit (Qiagen, Germany) and the NanoDrop2000 spectrophotometer was used to detect the concentration and purity of DNA samples.

#### 2.1.2 Whole exome sequencing

The DNA library was generated based on the Illumina standard protocol. High-throughput sequencing was performed using the NovaSeq platform (Illumina, USA) with PE150 (paired-end sequencing, 150 bp read), with an average depth coverage of 100-fold. The sequence data were aligned with the human reference genome GRCh37/hg19 using Sentieon BWA v0.7.15 software. SAMtools v1.9 and Sentieon GATK software v4.1.4.0 were used to detect SNPs and short indels. Variants were annotated using the Alamut-Batch standalone version v1.9 software (Interactive Biosoftware, France) and searched for known disease-causing variants using HGMD® Professional. All variants were manually interpreted according to the American College of Medical Genetics and Genomics (ACMG) scoring system and recommendations from the ClinGen Sequence Variant Interpretation (SVI) Working Group.

#### 2.1.3 Sanger sequencing validation

Sanger sequencing was used to validate the signifcant variants identifed by exome sequencing. Briefy, exons containing the variants were amplifed by PCR and both strands were sequenced using ABI 3730xl sequencerr (Applied Biosystems, Foster City, CA, USA), and sequence chromatograms were analyzed using Chromas 2.6.5 sofware (Technelysium,Brisbane, Australia). Primers (Table 1) were designed by Primer 5 software (Biosoft Intemational, Palo Alto, USA).

**Table 1:** Primer sequences of exon4 and exon10 of GLB1.

Exon		Sequence (5′-3′)
Exon4	Forward	AGCTCAAATACCCCTTGTCCC
	Reverse	CCCAGCCTTCAAAATACCTCC
Exon10	Forward	TCCCAAAGAACTTGTCCCAGAG
	Reverse	TCTCCCATCACCCTACCTCTC

#### 2.1.4 Variant pathogenicity analysis

For variant sites, the online tools UniProt, polyphen-2 and mutationtaster were used to analyze their conservation and pathogenicity. Besides, three-dimensional structure models of protein for each mutation were constructed. Modeling was performed using Alphafold, and Pymol was used to visualize the effects of altered residues on the protein structures.

#### 2.1.5 Construction of wild-type and mutant GLB1 expression plasmids

The wild-type GLB1 cDNA (GenBank: NM_000404.3) was synthesized and cloned into the pcDNA3.1 plasmid (Invitrogen, Carlsbad, California). Site-directed mutagenesis was performed to introduce the c.446C>T, c.1058_1059delinsAA, c.1058T>A, c.1059C>A and c.495_497delTCT mutations into the wild-type GLB1 cDNA and confirmed by sequencing. A reported pathogenic mutation c.495_497delTCT was performed as positive control (13).

#### 2.1.6 Transient transfection

293T cells were cultured in DMEM supplemented with 10% fetal bovine serum, 100 U/ml penicillin and 100 μg/ml streptomycin at 37℃ and 5% CO_2_. Cells were transfected with 2 ug wild-type or mutant DNA using Lipofectamine 3000 (Invitrogen, Carlsbad, CA) according to manufacturer's manual. After 48 h incubation at 37℃, cells were washed twice with PBS and scraped.

#### 2.1.7 Enzyme activities assay

The harvested 293T cells were sonicated in ddH_2_O and then protein concentrations were determined using BCA Protein Assay Kit (Beyotime, Shanghai, China). The β-galactosidase activities were measured using the synthetic substrates, 4-methylumbelliferyl -β-D-galactopyranoside (Sigma, St. Louis, MO). The enzyme activities of mutants were expressed as the percentages of wild-type enzyme activity.

### 2.2 Statistical analysis

All experiments were repeated three times independently. The difference between the wild-type enzyme activities and the individual mutant enzyme activities were analyzed by GraphPad Prism 5 (GraphPad Software Inc., CA, USA) using Student's *t*-test. *P* values of ≤0.05 were considered statistically significant.

## 3 Results

### 3.1 Mutation analysis of GLB1 gene

**Figure 2:**
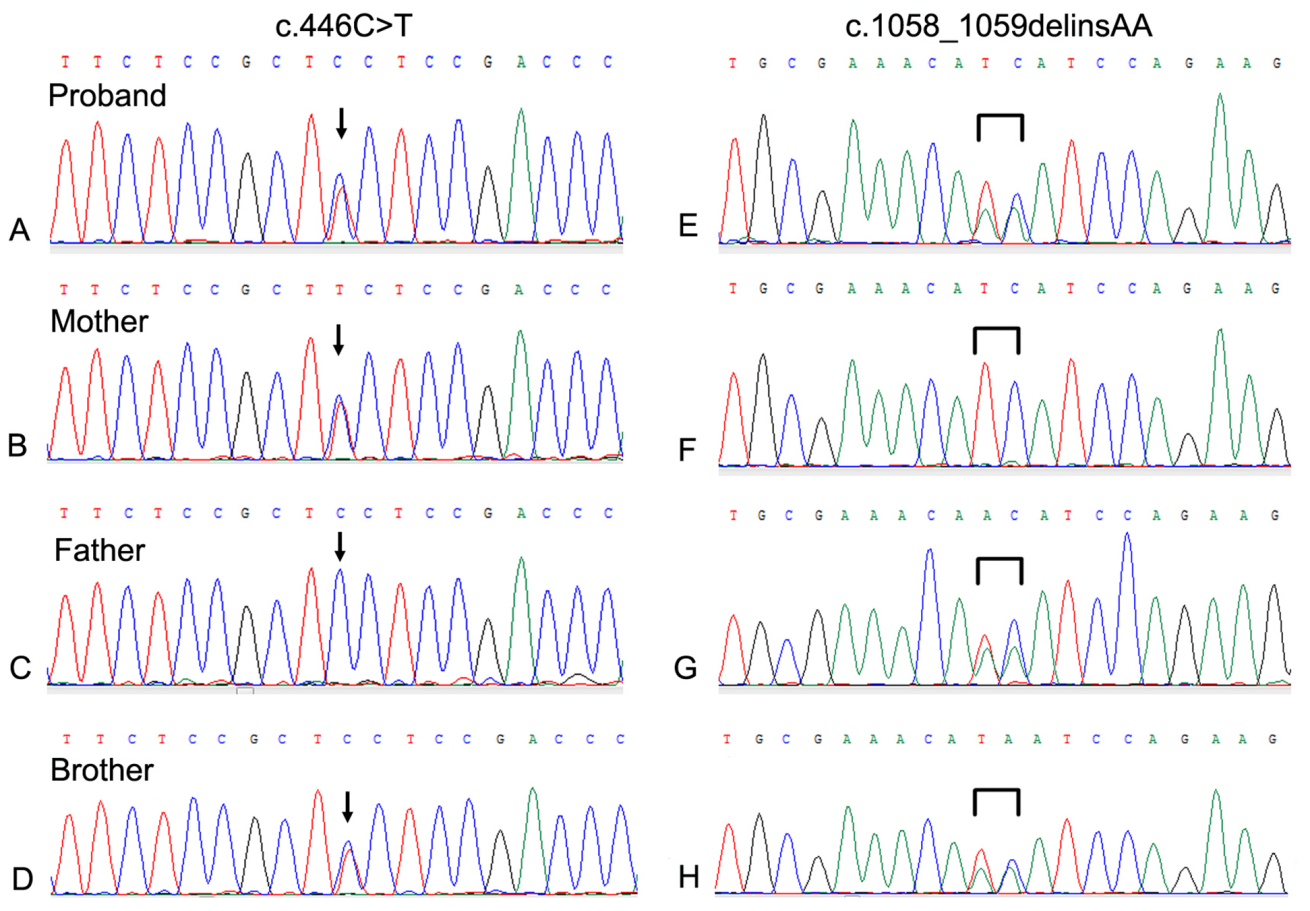
Sanger sequencing of the neighboring regions of the mutations in the GLB1 gene of the family. **(A–D)** The c.44C>T mutation of the proband and his brother was derived from the mother; **(E–H)** The c.1058_1059delinsAA mutation of the proband and his brother was derived from the father.

**Figure 3:**
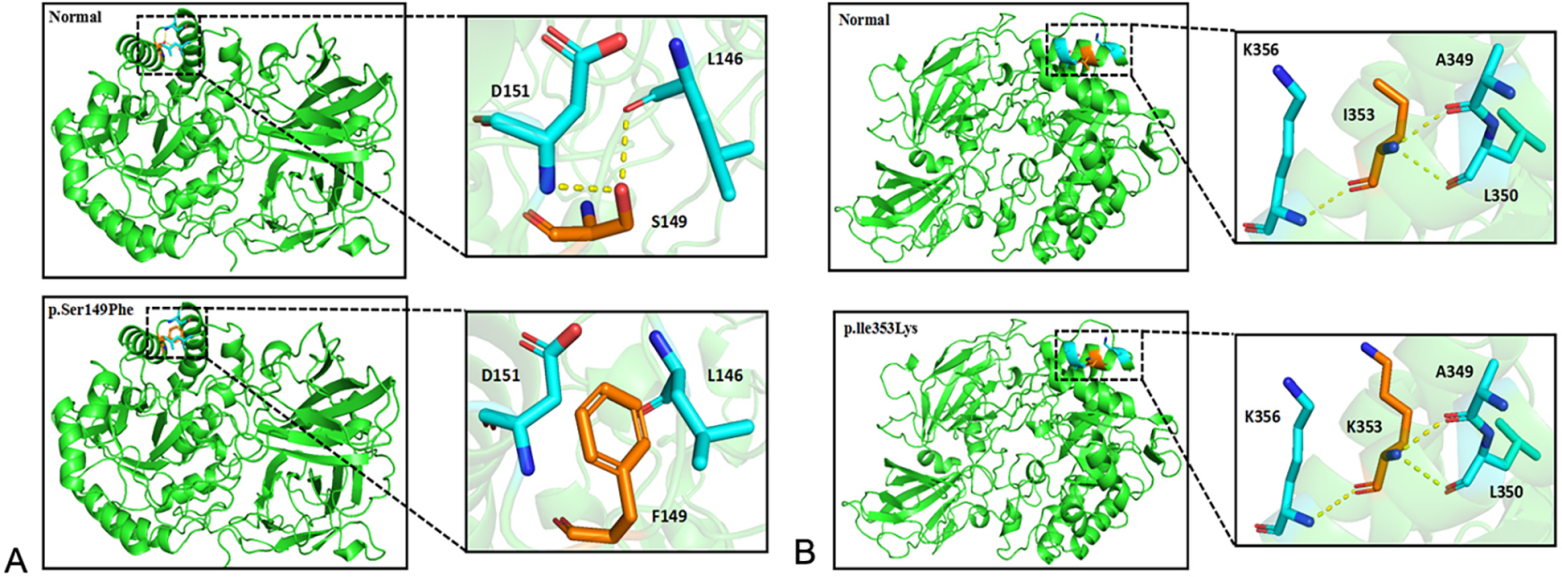
Three dimensional structure analysis of GLB1 mutant protein. **(A)** After the p.S149F mutation occurs, the interaction force between amino acids 149 and 146 and 151 disappears or weakens; **(B)** After the p.l353K mutation occurs, the number of hydrogen bonds between amino acid 353 and adjacent amino acids does not change.

**Figure 4:**
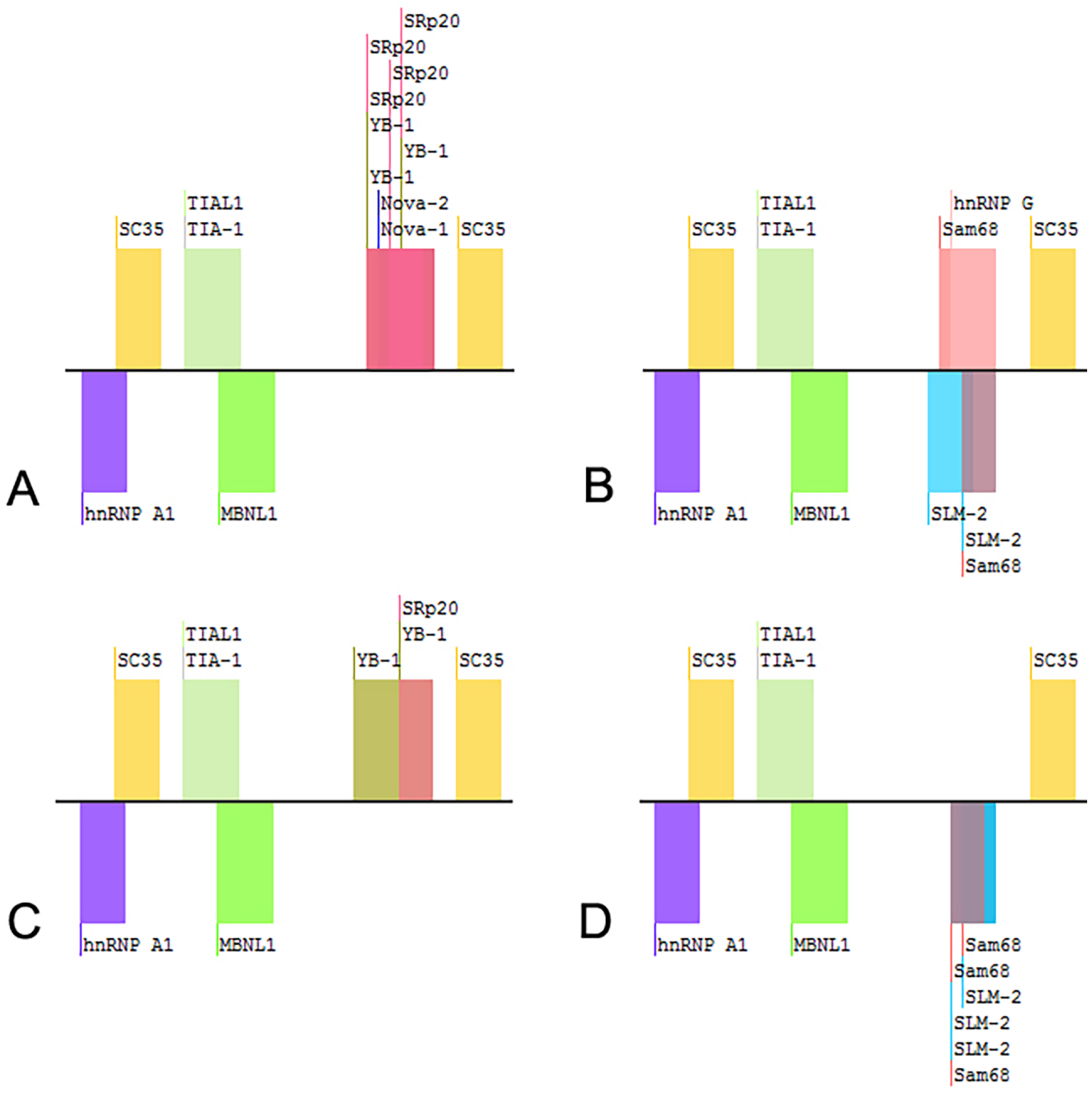
Splicing factors related to the variants of GLB1 gene. **(A)** wild-type; **(B)** c.1058_1059delinsAA; **(C)** c.1058T>A; **(D)** c.1059C>A.

WES was performed on the proband. Two GLB1 gene mutations including a reported missense mutation (14), c.446C>T(p.S149F), in exon 4 and a novel deletion-insertion mutation, c.1058_1059delinsAA (p.I353K) in exon 10 were identified. Among them, the c.446C>T mutation originates from the mother, while c.1058_1059delinsAA from the father. The mutation sites were confirmed by Sanger sequencing (Figure 2). Moreover, mutations of the brother were same as that of the proband. Analysis of three-dimensional structure of protein suggested that this variant does not alter the number of hydrogen bonds between p.Ile353 and adjacent amino acid residues (Figure 3). In addition, splicing factor analysis using SpliceAid2 database indicated that multiple splicing factors including splicing enhancer such as SRp20, YB-1 and hnRNP G and splicing silencer such as Sam68 and SLM-2 were affected by the mutations (Figure 4).

### 3.2 Pathogenicity analysis of variant loci

**Figure 5:**
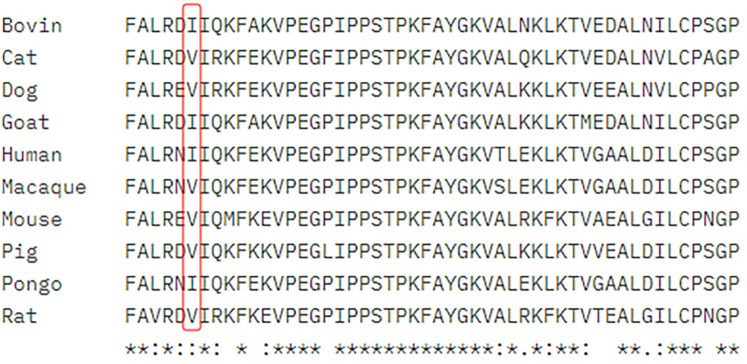
Alignment of the GLB1 homologs of ten species around the p.I353K variant. *Total sequence homology; Very high homology; High homology.

The mutation p.S149F has been reported as a pathogenic variant, described as DM in the HGMD database. The allele frequency of this variant in the total population is less than 0.01% (2/249264), with no homozygous reports in the gnomAD database. It has the highest detection frequency in the East Asian population, at 0.006% (1/17976). The c.1058_1059delinsAA variant has not been reported in the literature and disease databases. Alignment of the GLB1 sequences from human, mouse, rat, bovin, cat, dog, goat, pig, macaque and pango revealed that p.Ile353 is high level of homology throughout these species (Figure 5). According to the ACMG guidelines, it is recommended that this variant was rated as a variant of uncertain significance.

### 3.3 β-galactosidase activity of transfected plasmids

**Figure 6:**
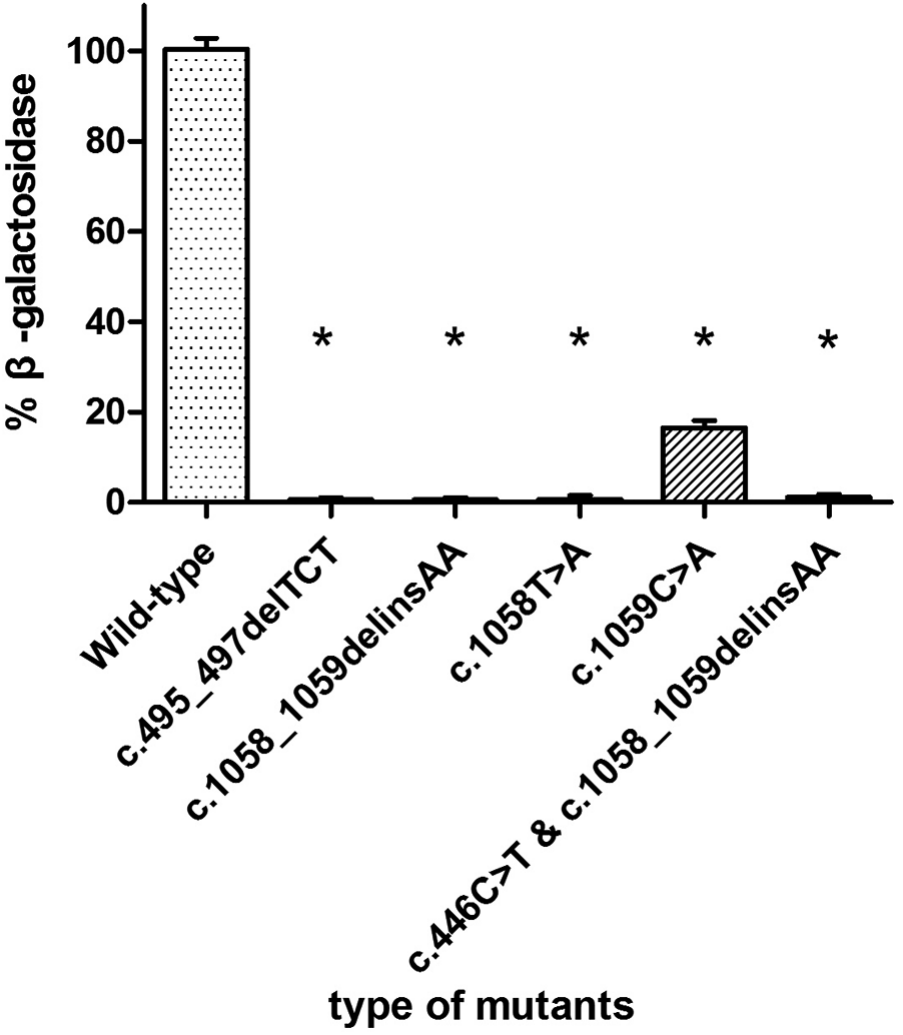
β-galactosidase activity in 293T cells transfected with wild-type and mutant plasmids.*P < 0.05.

The enzyme activity of each mutant was compared with that of transfected wild-type GLB1 gene. As shown in Figure 6, the positive control c.495_497delTCT mutants retained the β-galactosidase activity at 0% of wild-type GLB1. The mutants c.1058_1059delinsAA, c.1058T>A and c.1059C>A retained β-galactosidase activity at 0%, 0% and 16.67% of wild-type GLB1, respectively (*P* < 0.05). In addition, the mutant containing both c.446C>T and c.1058_1059delinsAA mutations also retained 0% of the wild-type GLB1 β-galactosidase activity (*P* < 0.05).

## 4 Discussion

According to onset age, disease manifestations and genetic analysis, both of our patients were classified as late-infantile form of GM1 gangliosidosis. As previously mentioned, the clinical symptoms of GM1 gangliosidosis infantile form manifest in the first three months of life. However, patients with late-infantile or adult form have normal neurodevelopment before late infancy or late childhood. Therefore, molecular diagnosis should be made early in presymptomatic cases with positive family history. For late onset disease types, enzyme replacement therapy or cell therapy can be used in advance. It is difficult for clinicians to make a correct diagnosis because the accumulated metabolite GM1 ganglioside of the disease cannot be identified by routine metabolic examination (15, 16). In recent years, high-throughput sequencing technology has played an important role in the etiological diagnosis of genetic diseases (17, 18). In particular, WES can detect the pathogenic variant sites of multiple genes and exons in the human genome at one time, which is essential for the precise diagnosis of genetic diseases. In this study, we performed WES on a patient who showed significant developmental regression and found p.S149F and c.1058_1059delinsAA compound heterozygous mutation in the GLB1 gene was inherited from his mother and father respectively. The proband's older brother had the same mutation sites, which provided a reliable basis for the diagnosis of GM1 gangliosidosis.

Previous studies have shown that there are regional differences in the subtypes and gene mutation characteristics of the disease. Infantile form is mainly common in the United States, Europe and Brazil (19), while adult form is more common in Japan, which probably be related to p.I15T mutation (20). Several studies have confirmed infantile and late-infantile forms are the most common phenotypes in China (13, 21). Because most patients' mutations are compound heterozygous, the same pathogenic variants are identified in more than one phenotype, making it difficult to establish the correlation between genotype and phenotype. As a lysosomal hydrolase, β-galactosidase can cleave the terminal β-galactose in gangliosides or other glycolipids, thereby reducing the accumulation in the body. β-galactosidase includes TIM barrel domain with catalytic function, β-domain 1 and β-domain 2, which are composed of 677 amino acid residues (22). A study (23) indicated that most mutations located in the core region of the protein structure, especially those in the TIM barrel domain responsible for catalysis, have a significant impact on the structure and stability of the protein encoded by this gene. The amino acid residue changes at position 149 and 353 caused by the p.S149F and c.1058_1059delinsAA variants are located in the TIM barrel domain of GLB protein, thus affecting the GLB protein activity and causing GM1 gangliosidosis.

GM1 gangliosidosis often shows extensive myelination abnormalities in the brain, symmetrical abnormal signals in the basal ganglia and thalamus, brain atrophy, and multiple bone dysplasia in imaging examination (24). However, the imaging findings are not specific, and different patients have diversified manifestations, which will change with the progression of the disease. MRI examination of our patient suggested that the cerebral white matter was hypoplastic, which was consistent with the imaging findings of GM1 gangliosidosis. In addition, some studies (8, 25) revealed that the increase of AST level can be used as a biomarker for the early diagnosis of GM1 gangliosidosis, but in this case, we found that except for the mild increase of blood ammonia and lactate, no obvious abnormalities were observed in other laboratory tests, indicating that the increase of AST is not universal for the diagnosis of this disease.

Two variants were detected in this study, among which the missense mutation p.S149F was a reported pathogenic variant, and c.1058_1059delinsAA was a novel mutation which was predicted to be a variant of uncertain significance by prediction software. Indel mutation is a genetic mutation caused by the insertion or deletion of a segment of DNA that is often less than 50 bp, into an organism's genome. A total of eight indel mutations with different pathogenicity of GLB1 gene have been decribed (Table 2). The three-dimensional structure of a protein is determined by the sequence of amino acid residues, and any amino acid residue will have an impact on the overall physical properties of the protein. Similar amino acid residue sequences have analogous three-dimensional spatial conformations, which is the theoretical basis of protein three-dimensional structure prediction. We used molecular modeling to predict the conformational change of the protein, thereby interpreting the c.1058_1059delinsAA may alter the enzymatic activity of β-galactosidase. Although the number of hydrogen bonds were not changed, p.Ile353 is proven to be highly homologous among common mammals. SpliceAids2 database was used to estimate the effect of c.1058_1059delinsAA mutation at the level of the target sequences of the RNA-binding proteins that determine the pattern of mRNA splicing. It indicated that SRp20, YB-1 and Sam68 may be the key splicing factors related to the c.1058_1059delinsAA mutation. SRp20 protein is a member of the SR(Ser-Arg rich) protein family, which has a highly conserved serine/arginine sequence. This protein plays a role in the nucleus and participates in the splicing process of pre-mRNA, that is, removing introns from pre-mRNA and connecting exons to produce mature mRNA molecules. We speculated this mutation may affect the splicing of GLB1 by SRp20, thus reducing the activity of β-galactosidase, but the exact molecular mechanism still need to be further researched. In functional studies, low enzyme activity (0% of wild-type) was detected in 293T cells transfected with a plasmid encoding c.1058_1059delinsAA. These results strongly suggested c.1058_1059delinsAA should be classified as pathogenic or likely pathogenic variant.

**Table 2:** Indel mutations of GLB1 gene on the clinVar website.

Nucleotide change	Amino acid change	Variant type	Classification
c.1724_1725delinsTG	p.G575V	Missense variant	Uncertain significance
c.1188_1189delinsCT	p.P397S	Missense variant	Likely pathogenic
c.1071_1073delinsGG	p.F357fs	Frameshift variant	Likely pathogenic
c.902_914+17delinsAGGCAAGTATATACTTGCC	–	Splice donor variant	Likely pathogenic
c.914+15_914+16delinsAGTGTGAACTTGTGAGTGTTT	–	Intron variant	Uncertain significance
c.817_818delinsCT	p.W273l	Missense variant	Pathogenic/Likely pathogenic
c.137_138delinsCC	p.Q46P	Missense variant	Uncertain significance
c.76-9_76-8delinsAA	–	Intron variant	Likely benign

In order to explore the impact of single base variations in c.1058_1059delinsAA, we also conducted splicing site analysis and enzyme activity determination of mutant plasmids. The enzyme activity caused by c.1058T>A and c.1059C>A, both of which were not reported, was significantly reduced although c.1059C>A represented as a synonymous mutation.

At present, symptomatic treatment is the main therapy for GM1 gangliosidosis, and there is no effective radical treatment. The difficulty lies in the effective removal of ganglioside accumulated in the nervous system. Present study (26) has indicated that the fusion protein of ricin toxin B chain (RTB) and β-galactosidase can retain both lectin activity and β-galactosidase activity, deliver the enzyme to cells to achieve lysosomal processing, and mediate GM1 ganglioside substrate clearance at the cellular level, which can be used as an effective alternative enzyme for GM1 gangliosidosis. The clinical trials of gene therapy and hematopoietic stem cell transplantation in lysosomal storage diseases also provide new strategies for the treatment of GM1 gangliosidosis (27). For families with probands, it is necessary to recommend them for relevant genetic counseling and prenatal diagnosis before giving birth again, which is conducive to reducing the occurrence of birth defects.

## 5 Conclusion

We detected compound heterozygous mutations in GLB1 gene by WES in a Chinese family with GM1 gangliosidosis presenting with late-infantile form, in which c.1058_1059delinsAA is a novel mutation. In cultured 293T cells, this mutation causes residual β-galactosidase activity to be 0% of normal activity. Together with the c.446C>T mutation, it affects GLB protein activity and leads to GM1 gangliosidosis. Our study expands the GLB1 gene mutation spectrum of GM1 gangliosidosis, emphasizes the importance of WES in evaluating patients with undiagnosed genetic diseases, and provides a new basis for genetic counseling.

## Data Availability

The original contributions presented in the study are included in the article/Supplementary Material, further inquiries can be directed to the corresponding author.
